# B-13 progenitor-derived hepatocytes (B-13/H cells) model lipid dysregulation in response to drugs and chemicals

**DOI:** 10.1016/j.tox.2017.05.014

**Published:** 2017-07-01

**Authors:** Alistair C. Leitch, Philip M.E. Probert, James A. Shayman, Stephanie K. Meyer, George E.N. Kass, Matthew C. Wright

**Affiliations:** a Institute of Cellular Medicine, Medical School, Newcastle University, Newcastle Upon Tyne, UK; b Department of Internal Medicine, University of Michigan Medical School, University of Michigan, Ann Arbor, MI, USA; c European Food Safety Authority, Parma, Italy

**Keywords:** ADFP, adipose differentiation-related protein, B-13, AR42J-B-13, B-13/H, AR42J-B-13 cells following treatment with 10 nM dexamethasone for 14 days, CYP, cytochrome P450, DAPI, 4’6’-diamino-2-phenylindole, DEX, dexamethasone, DTT, dithiothreitol, LAMP1, lysosome-associated membrane protein 1, LPLA2, lysosomal phospholipase A2, LXR, liver X receptor, NASH, non-alcoholic steatohepatitis, PB, phenobarbital, PCN, 5-pregnen-3β-ol-20-one-16α-carbonitrile, PEI, polyethylenimine, PXR, pregnane X-receptor, RXR, retinoid X-receptor, LXR, AR42J-B13, Steatosis, Phospholipidosis, T0901317, In vitro and alternatives

## Abstract

Lipid dysregulation is a common hepatic adverse outcome after exposure to toxic drugs and chemicals. A donor-free rat hepatocyte-like (B-13/H) cell was therefore examined as an in vitro model for investigating mechanisms. The B-13/H cell irreversibly accumulated triglycerides (steatosis) in a time- and dose-dependent manner when exposed to fatty acids, an effect that was potentiated by the combined addition of hyperglycaemic levels of glucose and insulin. B-13/H cells also expressed the LXR nuclear receptors and exposure to their activators – T0901317 or GW3965 – induced luciferase expression from a transfected LXR-regulated reporter gene construct and steatosis in a dose-dependent manner with T0901317. Exposing B-13/H cells to a variety of cationic amphiphilic drugs – but not other hepatotoxins – also resulted in a time- and dose-dependent accumulation of phospholipids (phospholipidosis), an effect that was reduced by over-expression of lysosomal phospholipase A2. Through application of this model, hepatotoxin methapyrilene exposure was shown to induce phospholipidosis in both B-13 and B-13/H cells in a time- and dose-dependent manner. However, methapyrilene was only toxic to B-13/H cells and inhibitors of hepatotoxicity enhanced phospholipidosis, suggesting phospholipidosis is not a pathway in toxicity for this withdrawn drug. In contrast, pre-existing steatosis had minimal effect on methapyrilene hepatotoxicity in B-13/H cells. These data demonstrate that the donor free B-13 cell system for generating hepatocyte-like cells may be employed in studies of fatty acid- and LXR activator-induced steatosis and phospholipidosis and in the dissection of pathways leading to adverse outcomes such as hepatotoxicity.

## 1 Introduction

The liver is a common target organ for drug- and chemical-mediated toxicity and the hepatocyte is most frequently the cell that is injured, in part, because of its active transport and first pass metabolism of drugs and chemicals (Wallace et al., 2008). The hepatocyte also plays a prominent role in the control of body’s metabolism and this includes lipid homeostasis. Hepatocytes synthesise triglycerides; synthesise lipoprotein components used to transport lipids throughout the body and are capable of active cholesterol and bile acid synthesis (Nguyen et al., 2008). Hepatocytes are also active in fatty acid oxidation, notably, through an up-regulation of peroxisomal fatty acid oxidation (Chamouton and Latruffe, 2012). A dysregulation in lipid homeostasis in hepatocytes is therefore often observed in the liver as an early response to exposure to toxic drugs and chemicals (Klaunig et al., 2003).

The B-13 cell line – originally cloned from the rat exocrine pancreatic cancer cell line AR42J (Mashima et al., 1996) – could offer a potential route to delivering a cost-effective, simple solution to the production of functional hepatocytes in vitro (for a full description of its derivation, see Probert et al., 2015). The B-13 cell is readily expandable in simple culture medium, does not require an extensive array of recombinant growth factors to retain its phenotype nor require sub-culture from non-progenitor cell types (such as fibroblasts) in its expansion, in contrast to pluripotent stem cells (Probert et al., 2015). Furthermore, B-13 cells do not require an extensive array of recombinant growth factors and/or other signalling inhibitor treatments to direct them to become hepatocytes. Exposure to a single glucocorticoid hormone is all that is required (Shen et al., 2000, Wallace et al., 2011). Critically, in contrast to stem cell-derived hepatocyte-like cells which generally only form a foetal level of hepatic differentiation, B-13/H cells express many adult hepatocyte genes at adult hepatocyte levels (Marek et al., 2003, Fairhall et al., 2013a, Fairhall et al., 2016). With respect to xenobiotic metabolism, B-13/H cells express functional CO-spectrophotometrically-detectable levels of many cytochrome P450s including Cyp1a1, Cyp2a1/2, Cyp2c6, Cyp2c11, cyp2c13, Cyp3a, Cyp3a2, Cyp3a9 and Cyp7a1 (Probert et al., 2015). A variety of phase 2 genes (sulfotransferases, UDP glucuronyl transferases, glutathione-S-transferases) have also been shown to be functional (Probert et al., 2015, Probert et al., 2016). A further advantage of B-13/H cells over other potential hepatocyte models such as stem cell derived hepatocytes, is that for reasons unknown, B-13/H cells remain differentiated for several weeks despite their culture on simple plastic cultureware. Primary hepatocytes and stem cell-derived hepatocyte-like cells (once driven toward a hepatocyte phenotype) still then undergo de-differentiation (Wallace et al., 2010a). The B-13 cell line – being freely available – therefore provides a cost-effective, reliable and repeatable model system in which liver functions may be studied. Although a number of studies have been completed with regard to B-13/H cells, xenobiotic metabolism and hepatoxicity, lipid dysregulation has not been investigated.

Steatosis defines primarily an accumulation of triglycerides and is an early adverse response to injury by hepatoxins such as carbon tetrachloride (Weber et al., 2003) or ethanol (Mathews et al., 2014, Louvet and Mathurin, 2015). It has been considered to be a relatively benign reversible response to some toxic stresses such as alcohol exposure. However, more recently it has become clear that it may be a critical early event in alcoholic liver disease pathogenesis and in non-alcohol dependent liver diseases such as non-alcoholic steatohepatitis (NASH) (Day, 2012).

The liver X receptor (LXR) is a member of the nuclear receptor gene superfamily of ligand-activated transcription factors (Jakobsson et al., 2012). Two closely related genes have been identified, LXRα and LXRβ. LXRβ is widely expressed whereas LXRα expression is restricted and highest in the liver. The LXRs are activated by oxysterols and have been considered as drug targets for treatment of hypercholesterolaemia. However, LXR activator drugs also have the effect of promoting liver steatosis (Repa et al., 2000, Cha and Repa, 2007).

Phospholipidosis is a lysosomal storage disorder characterised by accumulation of phospholipids in tissues, such as the liver (Shayman and Abe, 2013). Phospholipidosis is also seen in other tissues such as the kidney and lung and is a common response to most typically cationic amphiphilic drugs. The precise mechanism(s) which lead to accumulation of phospholipids and whether this response is a critical event in the mechanism of toxicity of causative drugs remain unclear (Shayman and Abe, 2013).

In this paper, B-13/H cells have been examined for their ability to re-capitulate lipid dysregulation in response to exposure to a variety of drugs and/or metabolic stresses that cause steatosis and phospholipidosis. We demonstrate that steatosis may be induced in B-13/H cells through exposure to high concentrations of fatty acids; that B-13/H cells express transcriptionally functional LXRα; that B-13/H cells are susceptible to steatosis after treatment with LXR activators and that exposing B-13/H cells to drugs which cause phospholiposis results in a dose- and time-dependent increase in phospholipid accumulation. Methapyrilene is a histamine H1 antagonist that was withdrawn after it was shown to cause liver tumours in rats (Reznik-Schüller and Lijinsky, 1981, Fischer et al., 1983). Methapyrilene also causes periportal liver injury in rats through a mechanism that requires metabolic activation (Ratra et al., 1998a). Since the chemical structure of methapyrilene shows all the attributes of a chemical that causes phospholidosis, the B-13-based model was applied to investigate this effect for the first time and to determine any role in hepatotoxicity.

## 2 Materials and methods

### 2.1 Plasmid constructs

The LXR responsive reporter gene construct, TK-LXRE-LUC (Willy et al., 1995), was generously provided by Prof David Mangelsdorf, Howard Hughes Medical Institute, University of Texas Southwestern Medical Center, Dallas, TX 75390-9050. An expression construct encoding the human lysosomal phospholipase A2 (LPLA2, also known as PLA2G15 or LYPLA3) cDNA sequence has been described previously (Hiraoka et al., 2002). All other materials were purchased from Sigma-Aldrich (Poole, UK) unless otherwise stated.

### 2.2 B-13 cell culture, treatments and transfection

B-13 cells (originally obtained from prof David Tosh, Bath University, UK) were routinely cultured in low glucose (1 g/l) Dulbecco’s Modified Eagle’s Medium (DMEM) containing 10% (v/v) fetal calf serum (FCS), 100 units/ml penicillin, 100 μg/ml streptomycin and 0.584 g/l l-glutamine. Cells were incubated at 37 °C at 5% CO_2_ in air in a humidified incubator and sub-cultured every 2–3 days by trypsinization (1:3 split) or differentiated – when the cells had reached approximately 40–50% confluency – to B-13/H cells via treatment with 10 nM dexamethasone (DEX) for 14 days, with media changes every 2–3 days (Marek et al., 2003). Hepatic phenotype (characterised by a marked phenotypic change) was routinely confirmed through expression of Cyp2e1 transcripts by RT-PCR (see below). Cyp2e1 has been shown to a sensitive marker of B-13 and both rodent and human pancreatic tissue conversion to an hepatocyte-like phenotype (Wallace et al., 2009, Wallace et al., 2010b, Fairhall et al., 2013b). Fatty acids (linoleic and oleic acid) were dissolved in ethanol to give a stock with a final concentration of 200 mM each. Fatty acid-free bovine serum albumin was dissolved in serum-free DMEM to give a final concentration of 0.8 mM. The fatty acids were then added to the medium to give a final concentration of 2 mM fatty acids (total fatty acid:BSA ratio = 2.5:1). BSA (Sigma) and ethanol were added to controls. All other chemical compounds were added to media from 1000 fold molar concentrated DMSO vehicle-solvated stocks (controls receiving 0.1% (v/v) DMSO), unless otherwise stated.

For reporter gene studies, B-13 cells seeded in 24 well plates were transfected with 0.25 μg DNA per well (pcDNA-empty or pcDNA-LPLA2 and a control plasmid (RL-TK, Promega) encoding the Renilla luciferase protein under the regulation of a constitutive thymidine kinase promoter) using polyethylenimine (PEI). Typically, for a 24 well plate, 12 μg of total DNA was added to 600 μl serum-free media and 72 μl of 1 mg/ml PEI solution and vortexed before being incubated at room temperature for 15 min. This mixture was then add to 3.6 ml of serum-free medium and 200 μl of this solution was added to each well and incubated at 37 °C for 2 h. After this period, a further 300 μl of serum-containing medium was then added and the cells further incubated for 22–46 h prior to further analyses.

### 2.3 Oil red stain and spectrophotometric quantification of triglyceride accumulation

Cells cultured in 24 well plates were washed twice with PBS (137 mM NaCl, 2.7 mM KCl, 10 mM phosphate, pH 7.4), fixed with 3.5% (w/v) formaldehyde in PBS for 30 min and then washed 3 times with PBS. The cells were then incubated with oil red O solution (84 mg oil red O dissolved in 24 ml of isopropanol overnight at room temperature prior to filtration and addition of 18 ml of water, incubation overnight at 4 °C, double filtration and long term storage at 4 °C) at room temperature for 5 min. Cells were then washed with water and stored in PBS prior to image analysis. A quantitative analysis was then performed through addition of a fixed volume of isopropanol to extract bound lipid and removal of solvent for absorbance determination at 515 nm. Pre-treatment with isopropanol was employed to determine lipid-free blank readings. The protein content in each well after removal of isopropanol and washing with PBS using the Lowry method was used to normalise lipid content to protein content. Note, that an increase in phospholipidosis did not result in an increase in cell-associated oil red stain (see Supp. Fig. S1).

Fig. S1 Specificity of oil red and LipidTOX stains for steatosis and phospholipidosis respectively. B-13/H cells were untreated (control) or treated daily for 2 days with medium supplemented where indicated with 2 mM fatty acids, its vehicle control (BSA control) or at the indicated concentration of selected amphiphilic drugs and incubated for the detection of steatosis or phospholipidosis as outlined in the Methods section. Twenty four hours prior to fixation and analysis, cells for phospholipidosis detection were additionally incubated with LipidTOX. Cells were fixed and either stained with oil red or stained with DAPI and imaged (A) and then steatosis lipid accumulation quantified by extraction of oil red from stained cells and determination of absorbance at 515 nm (B) or LipidTOX and DAPI fluorescence determined (C). Data are the mean and standard deviation of 3 separate determinations from the same experiment, typical of 3 separate experiments, *significantly different (two tailed) from control treated cells using ANOVA (p < 0.05) followed by Bonferonni post hoc test.

### 2.4 Triglyceride determination

Cells (combined from 6 wells from a 6 well plate) or tissue (approx. 50–100 mg) were washed with ice-cooled PBS. Cells were scraped into PBS, quickly centrifuged to pellet the cells, the supernatant was discarded and cells were re-suspended in ice-cooled 20 mM Tris buffer pH 7.4 with 100 mM KCl. Tissue was dabbed dry and added to 1 ml of ice-cooled 20 mM Tris buffer pH 7.4 with 100 mM KCl. Samples were sonicated and an aliquot removed for protein analysis. One volume of the remaining homogenate was added to 10 vol of lipid CM extraction buffer (chloroform/methanol) (2:1 v/v) with 150 μM butylated hydroxytoluene. The samples were vortexed for 30 s, then centrifuged for 1 min at 13,000*g* and the upper organic layer retained in a fresh tube. Homogenates were subjected to a repeat extraction and the second organic extract combined with the first. Approximately 10 mg silica gel (Sigma)/ml organic extract was added to bind phospholipid and after pelleting and separation, organic extracts were evaporated at 37 °C under a stream of nitrogen. Lipids were then re-suspended in 50 μl CM extraction buffer prior to lipase treatment and glycerol determination using a kit supplied by Abcam (Cambridge, UK) or analysis by TLC.

### 2.5 Phospholipid staining and quantification of phospholipid accumulation

Phospholipid in cells cultured in 24 well plates was stained using LipidTOX (Thermofisher, UK) essentially according to the manufacturer’s instructions. Cells were then washed in PBS, fixed in 3.7% (w/v) formaldehyde in PBS for 30 min, washed in PBS and then incubated with 20 μM 4′6′-diamino-2-phenylindole (DAPI) for 10 min to stain DNA. Cells were then washed and stored protected from light in PBS at 4–8 °C prior to imaging using a Nikon Spinning Disk confocal microscope or fluorescence determination at 595 nm excitation/615 nm (LipidTOX) followed by 360 nm excitation/460 nm emission (DAPI) for each well using a fluorescence plate reader. The LipidTOX fluorescence was normalised to DNA using the DAPI fluorescence, thereby controlling for cell number/well. Note, that an increase in steatosis did not result in an increase in LipidTOX fluorescence, rather a slight decrease (see Supp. Fig. S1).

### 2.6 Reverse transcriptase PCR (RT-PCR)

Total RNA was isolated from cells using TRIzol (Invitrogen, Paisley, UK) following the manufacturer’s instructions. RT-PCR was performed on total RNA essentially as previously described (Haughton et al., 2006). The primer sequences are given in Supplementary Table S1.

Table S1

### 2.7 Western blotting

Western blotting was performed essentially as previously described (Marek et al., 2005). The antibodies were purchased from Abcam and were used at the dilution given in brackets:-rabbit anti-lysosome-associated membrane protein 1 (LAMP1, 1:1000); rabbit anti-CYP2E1 (1:3000); rabbit anti-amylase (1:3000); mouse anti-β actin (1:3000); rabbit anti-HA tag sequence (1:4000) and mouse anti-LXR (1:1000). The relevant anti-IgG horseradish peroxidise conjugated secondary antibody and ECL based chemiluminescent detection (GE Healthcare, Amersham, UK) was used for detection using X-ray film.

### 2.8 Immunocytochemistry

Cells were washed twice with PBS then fixed using 2% (w/v) formaldehyde/0.2% glutaraldehyde in PBS for 20 min prior to a brief wash with PBS and incubation with ice-cooled methanol on ice for 10 min. After a brief rinse with PBS, cells were permeabilised and blocked through incubation with 5% (v/v) FCS/0.1% (v/v) tween 20 in PBS for 20 min followed by 4 × 5 min washes with PBS. Anti-LAMP1 antibody (Abcam) was incubated at a dilution of 1/200 in 0.5% (v/v) FCS in PBS overnight at 4 °C, followed by washing and incubation with FITC-conjugated anti-rabbit IgG for 30 min. The cells were then washed 4 × 5 min in PBS, incubated with DAPI to stain DNA, washed in PBS and imaged using a Nikon Spinning Disk confocal microscope.

### 2.9 Statistics

The student’s two-tailed *T* test was used to determine significant difference between groups. Significance was achieved where p < 0.05. For comparison of multiple groups, ANOVA was carried out and where significant, differences between groups were determined using Bonferroni–Holm method. Where p < 0.05, a significant difference was assumed.

## 3 Results

### 3.1 B-13/H cells accumulate triglycerides in response to exposure to fatty acids

**Fig. 1:**
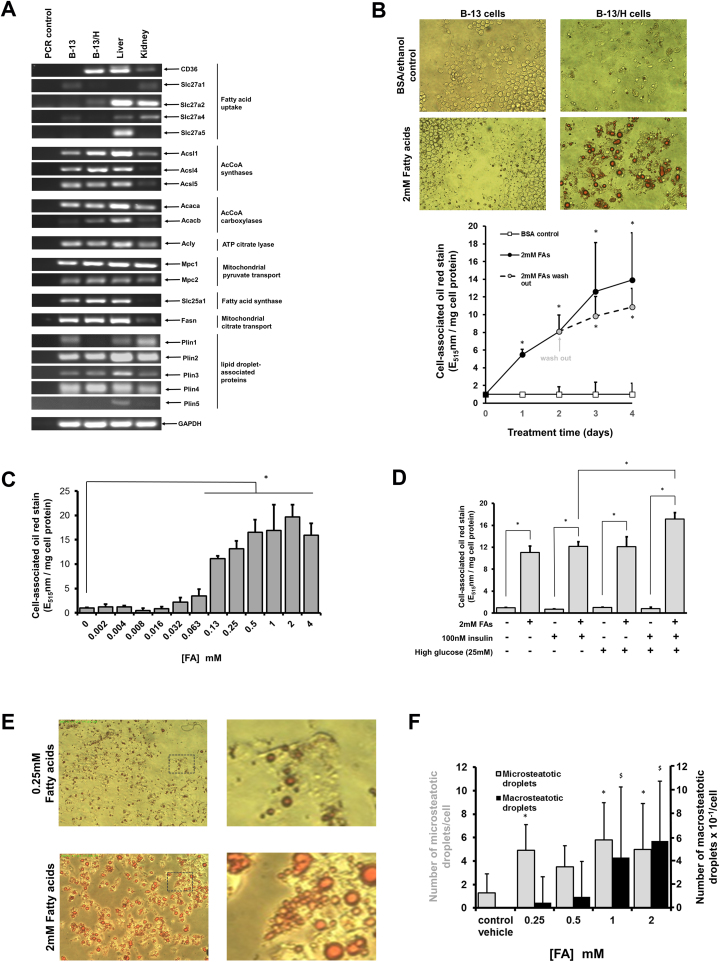
Exposing B-13/H cells to fatty acids results in a time- and dose-dependent irreversible micro and macrosteatosis. A, RT-PCR for the indicated transcripts in the indicated cell types or rat tissues. B (upper panels), B-13 and B-13/H cells were treated once without a medium change with medium supplemented where indicated with 2 mM fatty acids. After 3 days, cells were fixed and stained with oil red and imaged under bright field illumination; (lower panel) B-13/H cells were treated daily with medium supplemented where indicated with 2 mM fatty acids. At time zero, or 24 h after the last treatment, cells were fixed, stained with oil red and lipid accumulation quantified by extraction of oil red from stained cells and absorbance at 515 nm. Data are the mean and standard deviation of 3 separate determinations from the same experiment, typical of 3 separate experiments, *significantly different (two tailed) from control treated cells using ANOVA (p < 0.05) followed by Bonferonni post hoc test. C, Cells were treated once without a medium change with medium supplemented with the indicated concentration of fatty acids. After 3 days, cells were fixed, stained with oil red and lipid accumulation quantified by extraction of oil red from stained cells and absorbance at 515 nm. Data are the mean and standard deviation of 3 separate determinations from the same experiment, typical of 3 separate experiments, *significantly different (two tailed) from control treated cells using ANOVA (p < 0.05) followed by Bonferonni post hoc test. D, B-13/H cells were treated once without a medium change with medium supplementation as indicated. After 3 days, cells were fixed, stained with oil red and lipid accumulation quantified by extraction of oil red from stained cells and absorbance at 515 nm. Data are the mean and standard deviation of 3 separate determinations from the same experiment, typical of n separate experiments, *significantly different (two tailed) from cells treated identically but with fatty acids using the Student’s *T* test (p < 0.05). E, B-13/H cells were treated once without a medium change with medium supplemented with the indicated concentration of fatty acids. After 3 days, cells were fixed and stained with oil red and imaged under bright-field illumination. Right panels are exploded views of indicated region of left panels. F, quantification of the number of microsteatotic and macrosteatotic droplets in cells. Data are the mean and standard deviation number of droplets in 20 cells for each concentration, randomly selected by an investigator blinded to the treatments. *Significantly different (two tailed) from control treated cells using ANOVA (P > 0.95) followed by Bonferonni post hoc test. (For interpretation of the references to colour in this figure legend, the reader is referred to the web version of this article.)

B-13 and B-13/H cells express a variety of transcripts associated with fatty acid uptake and synthesis although there may be an increase in functional uptake capacity in B-13/H cells since there was an induction from undetectable levels of CD36 (also known as fatty acid translocase) in B-13/H cells (Fig. 1A). To determine whether B-13 and B-13/H cells were capable of sequestering fatty acids and synthesising and accumulating triglycerides, cells were exposed to 2 mM linoleic and oleic acid fatty acids. Both B-13 and B-13/H cells showed evidence of lipid accumulation as determined by oil red staining though this was more marked in B-13/H cells (Fig. 1B, upper panels) and was associated with a mean 16 fold increase in triglyceride content as determined by glycerol quantitation in phospholipid-free lipid extracts treated with lipase (Supp Fig. S2A). Lipid accumulation increased with incubation time and resisted significant reversal for at least 2 days when B-13/H cells were washed and incubated in medium free of fatty acids (Fig. 1B, lower panel). The increased variability of cell-associated oil red stain in un-washed cells from day 3 after loading was likely associated with stress associated with triglyceride accumulation since at time points beyond 4 days, significant cell death was noted (data not shown). Fig. 1C demonstrates that lipid accumulation in B-13/H cells was also dose-dependent. Addition of biologically relatively high levels of insulin (100 nM, typically levels in rat plasma are of the order 10^−10^ M, Bonner-Weir et al., 1983) or glucose (25 mM, a hyperglycaemic concentration) had no effect on lipid accumulation in B-13/H cells alone, or when incubated separately with 2 mM fatty acids. However, a combination of both insulin and glucose significantly increased the total accumulation lipids when also incubated with 2 mM fatty acids (Fig. 1D).

Fig. S2 Triglyceride accumulation in B-13/H cells. A, triglyceride content in B-13/H cells after incubation with 1mM fatty acids for 3 days. B, TLC analysis of lipids extracted from the indicated cells/tissue (triglycerides indicated by dotted circles; Ptdcholine = phosphatidylcholine).

Visual inspection of B-13/H cells suggested that the size of the lipid droplets could be classified as falling within two sizes when incubated with fatty acids (Fig. 1E). Incubating B-13/H cells at low concentrations of fatty acids resulted in primarily small droplet formation whereas at higher concentrations, both small and large droplets were formed (Fig. 1F).

Since susceptibility to non-alcoholic fatty liver disease (NAFLD) in man has been associated with a polymorphism in the Patatin-like phospholipase domain-containing protein 3 (PNPLA3) gene (I148M variant, rs738409, see Liu et al., 2014), the human and rat PNPLA3 cDNA sequences were aligned. Supp Fig. S3 demonstrates that rodent and human PNPLA3 N-terminal amino acid sequences show high homology and that the isoleucine at position 148 (which is replaced by a methionine in individuals with increased susceptibility to NAFLD) is conserved in both wild type proteins. A segment of the PNPLA3 transcript in B-13/H cells was amplified by RT-PCR, cloned and sequenced as outlined in the Methods section. Eleven clones were sequenced and all were shown to encode a wild type PNPLA3 transcript indicating, with greater than 95% confidence, that if susceptibility to NAFLD in the rat is similarly affected by this polymorphism, then B-13 cells have a homozygous wild type phenotype.

Fig. S3 The B-13 cells is homozygous wild type for the patatin-like phospholipase domain-containing protein 3 (Pnpla3) gene. A, CLUSTAL O (1.2.1) multiple sequence alignment (http://www.ebi.ac.uk/Tools/msa/clustalo/) of rat (rPNPLA3) and human (hPNPLA3) amino acid sequences, with the wild type isoleucine residue at position 148 indicated in red. “*”, a single, fully conserved residue; “:”, conservation between groups of strongly similar properties; “.”, conservation between groups of weakly similar properties; – no residue alignment. B, Alignment of CLUSTAL O (1.2.1) multiple sequence alignment of the wild type rat Pnpla3 cDNA sequence (wtPnpla3) and the predicted mutant Pnpla3 cDNA sequence (mutPnpla3) amplified by RT-PCR using the upstream (US) and downstream (DS) primers as indicated. Note, ATT codes for isoleucine (I) whereas ATG codes for methionine (M). Note, both ATC and ATA codons code for I and therefore the wild type protein. Therefore, restriction of PCR products with the endonuclease *Fat*I at this site indicates the existence of a mutant transcript, which in man, is strongly associated with NAFLD incidence (Anstee QM, Seth D, Day CP. Genetic Factors That Affect Risk of Alcoholic and Nonalcoholic Fatty Liver Disease. Gastroenterology. 2016 Jun;150:1728–1744). C, Typical sequencing data from RT-PCR product amplifying the region of the B-13 patatin-like phospholipase domain-containing protein 3 (PNPLA3) cDNA sequence.

These data therefore demonstrate that B-13/H cells accumulate lipid droplets when incubated with fatty acids in a time- and dose-dependent manner, with the morphological appearance of primarily microsteatosis at lower concentrations increasing to additionally macrosteatosis at higher concentrations. B-13/H cells also showed an increase lipid accumulation when high fatty acid levels were also co-incubated with both insulin and hyperglycaemic levels of glucose.

### 3.2 B-13/H cells have a functional LXR response

The LXR is a nuclear receptor that is activated by oxysterols and has been considered as a potential drug target for the treatment of hypercholesterolaemia (Bełtowski, 2008, Calkin and Tontonoz, 2012). LXR ligand activators such as T0901317 (Houck et al., 2004) and GW3965 (Kotokorpi et al., 2007) promote reductions in circulating cholesterol levels by inhibiting intestinal cholesterol absorption, stimulating cholesterol efflux from cells/transport to the liver and conversion to bile acids and biliary excretion (by the liver). However, these chemicals also promote steatosis in the liver as an adverse effect (Repa et al., 2000, Cha and Repa, 2007).

**Fig. 2:**
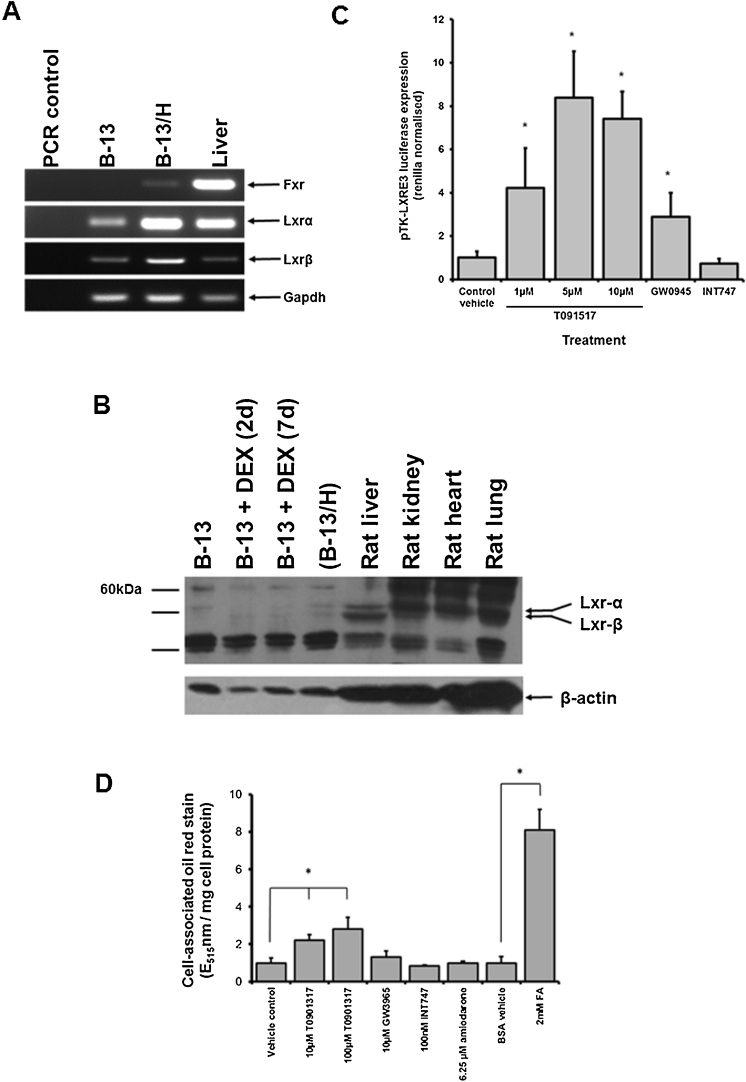
B-13/H cells express the LXR and accumulate lipids in response to treatment with the LXR activator T0901317. A, RT-PCR for the indicated transcripts in RNA isolated from the indicated cell/tissue. B, Western Blot for the indicated proteins in extracts from the indicated cell/tissue. Each lane contains 10 μg protein/lane. C, B-13/H cells were transfected with pTK-LXRE3 luciferase and RL-TK constructs as outlined in the Methods section and after 48 h, cells were treated with the indicated compound and luciferase and renilla activities determined after a further 24 h. Data are the mean and standard deviation of 6 separate determinations from the same experiment, typical of 3 separate experiments. D, B-13/H were treated once for 3 days with medium supplemented with the indicated concentration of compound. After 3 days, cells were fixed, stained with oil red and lipid accumulation quantified by extraction of oil red from stained cells and absorbance at 515 nm. Data are the mean and standard deviation of 3 separate determinations from the same experiment, typical of 2 separate experiments, *significantly different (two tailed) from control treated cells using ANOVA (P > 0.95) followed by Bonferonni post hoc test. (For interpretation of the references to colour in this figure legend, the reader is referred to the web version of this article.)

Fig. 2A demonstrates that B-13 and B-13/H cells express both LXRα and LXRβ transcripts with a significant increase in expression of both transcripts in the B-13/H phenotype. In contrast, the FXR nuclear receptor transcript (which regulates the expression of genes associated with the synthesis of bile acids from cholesterol) is only expressed at low levels in B-13/H cells (Fig. 2A). Fig. 2B indicates that LXR transcripts are translated to low (undetectable) levels in B-13/H cells when compared to rat hepatocytes. However, the LXR activators T0901317 and GW3965 readily transactivated an LXR regulated luciferase reporter construct suggestive of functional LXR transcriptional activity, whereas the FXR agonist INT747 (Pellicciari et al., 2002) had no effect (Fig. 2C). Exposing B-13/H cells to T0901317 resulted in significant lipid accumulation whereas the weaker LXR activator GW3965 increased levels but not to a statistically significant level (Fig. 2D). However, it has been reported that GW3965 induced ADFP (a protein residing at the surface of lipid droplets) in human but not in rat hepatocytes and that this accounts for a lack of steatosis with GW3965 in rat hepatocytes (Kotokorpi et al., 2007).

These data indicate that the adverse accumulation of lipids that occurs in response to LXR accumulation may be modelled in B-13/H cells although the response may be ablated due to low levels of LXR expression.

### 3.3 B-13/H cells accumulate phospholipids in response to exposure to cationic amphiphilic drugs

**Fig. 3:**
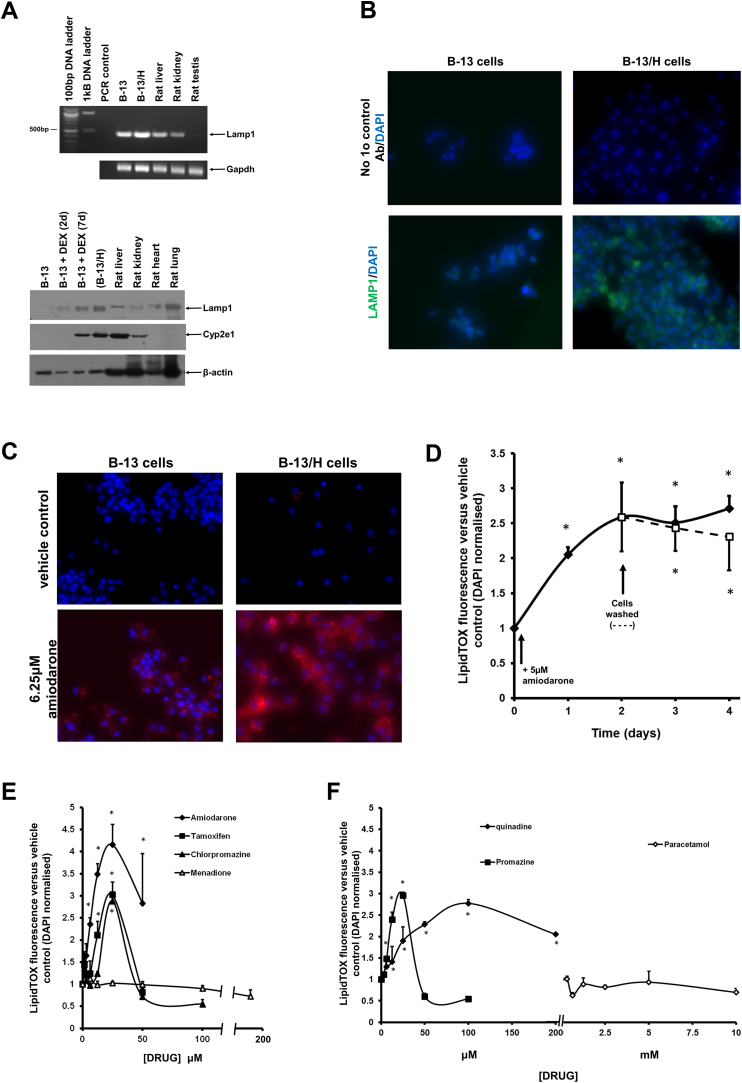
Exposure to cationic amphiphilic drugs results in phospholipidosis in B-13 and B-13/H cells. A, Upper panel, RT-PCR for the indicated transcripts in RNA isolated from the indicated cell/tissue; lower panel, Western Blot for the indicated proteins in extracts from the indicated cell/tissue. Each lane contains 10 μg protein/lane. B, immunocytochemical staining for LAMP1 (green) in B-13 and B-13/H cells with DAPI (blue) staining DNA. No 1° Ab control, identical staining procedure with the sole exception of incubation with primary anti-LAMP1 antibody. C, B-13 or B-13/H cells were treated once for 2 days with medium supplemented with 6.25 μM amiodarone. After 24 h, cells were additionally incubated with LipidTOX (red) and at 48 h cells were fixed and stained with DAPI (blue) prior to fluorescence imaging as outlined in Methods section. D, B-13/H cells were treated daily (with daily fresh medium changes) with 6.25 μM amiodarone. Twenty four hours prior to fixation and analysis, cells were also additionally incubated with LipidTOX. Cells were fixed, stained with DAPI and phospholipidosis quantified and normalised to DAPI fluorescence as outlined in Methods section. Data are the mean and standard deviation of 3 separate determinations from the same experiment, typical of 2 separate experiments, *significantly different (two tailed) from cells at time zero using ANOVA (p < 0.05) followed by Bonferonni post hoc test. E/F, B-13/H were treated once for 2 days with medium supplemented with the indicated concentration of drugs. After 24 h, cells were additionally incubated with LipidTOX (red) and at 48 h cells were fixed and stained with DAPI (blue) and phospholipidosis quantified and normalised to DAPI fluorescence as outlined in Methods section. Data are the mean and standard deviation of 3 separate determinations from the same experiment, typical of 2 separate experiments, *significantly different phospholipidosis (two tailed) from control treated cells using ANOVA (p < 0.05) followed by Bonferonni post hoc test. Note, cell viability as determined by MTT assay was significantly reduced compared to untreated cells at 50 μM amiodarone and at concentrations ≥50 μM tamoxifen, ≥50 μM tamoxifen, ≥100 μM chlorpromazine, ≥6.25 μM menadione and ≥50 μM promazine. (For interpretation of the references to colour in this figure legend, the reader is referred to the web version of this article.)

Phospholipidosis is a lysosomal storage disorder characterised by accumulation of intracellular phospholipids (Shayman and Abe, 2013). A large number of cationic amphiphilic drugs have been reported to induce phospholipidosis, suggesting that they share a causative target in cells. Fig. 3A and B demonstrates that B-13/H cells expressed readily detectable levels of the lysosomal-associated membrane protein 1 (LAMP1), a lysosomal marker protein, by RT-PCR, Western blotting and immunocytochemistry.

Fig. 3A suggest that whilst B-13 cells constitutively expressed Lamp1 mRNA, Lamp1 protein was not readily detectable. However, in response to DEX and conversion to B-13/H cells (as judged by expression of Cyp2e1), Lamp1 protein was induced to detectable levels and was present at levels higher than in rat liver (Fig. 3A, lower panel). Note that a variety of cytochrome P450s are expressed in B-13/H cells although this is not the case with the fatty acid metabolising Cyp4a genes which appear to contain several insertions and deletions in the locus (Probert et al., 2015). Thus, B-13/H cells respond to peroxisome proliferator activated receptor alpha ligands resulting in peroxisomal proliferation, but expression of Cyp4a1, Cyp4a2 and Cyp4a3 is not observed (Probert et al., 2015).

Treating both B-13 and B-13/H cells with the drug amiodarone, which has been reported to induce phospholipidosis in variety of cell types (Poucell et al., 1984, Pintavorn and Cook, 2008, Kapatou et al., 2010), induced phospholipidosis – most markedly in B-13/H cells (Fig. 3C) – a response that increased with exposure time and was essentially irreversible (Fig. 3D). Fig. 3E and F demonstrate that exposing B-13/H cells to a variety of different cationic amphiphilic drugs (amiodarone, tamoxifen, chlorpromazine, quinidine and promazine), but not structurally unrelated drugs (menadione, paracetamol) resulted in a dose-dependent increase in phospholipidosis that was reduced at higher (more toxic) concentrations.

### 3.4 The role of LPLA2 in B-13 cell phospholipidosis

The mechanism leading to the accumulation of phospholipids in cells exposed to cationic amphiphilic drugs has been proposed to be associated with an block in lysosomal phospholipase A2 (LPLA2) interaction with lysosomal membranes (and to its subsequent increased degradation), resulting in an inhibition of phospholipid turnover and therefore accumulation (Shayman and Abe, 2013).

**Fig. 4:**
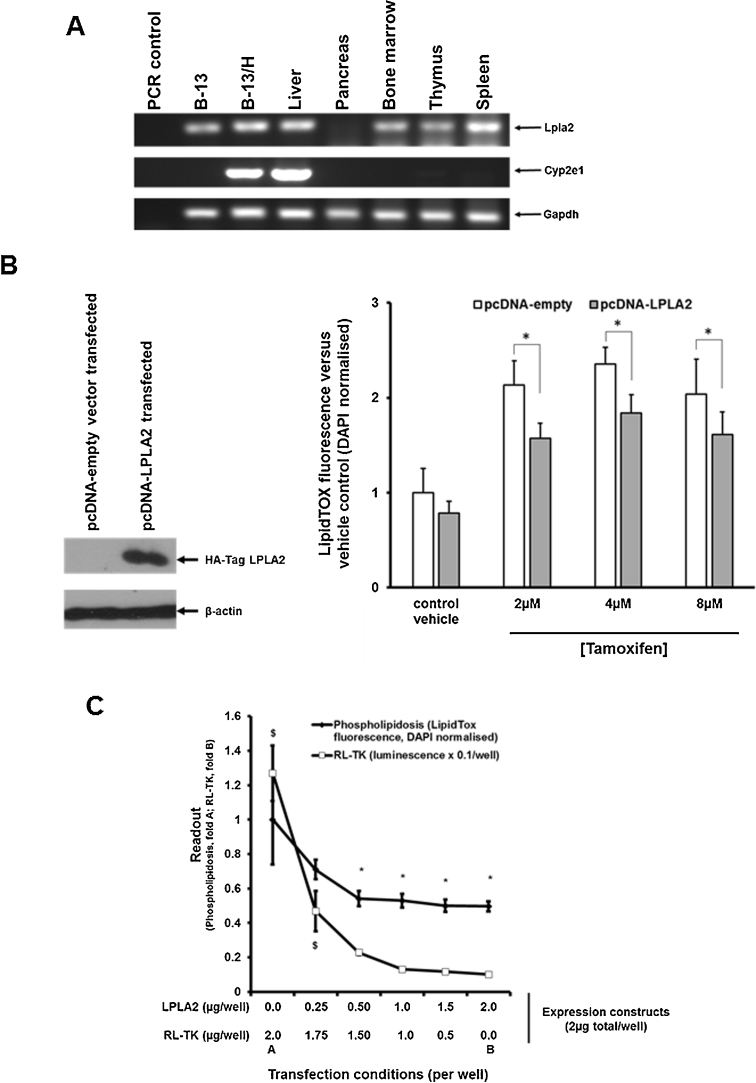
Human LPLA2 expression reduces tamoxifen-induced phospholipiosis in B-13 cells. A, RT-PCR for indicated transcripts in the indicate cell types or rat tissues. B, Western Blot in extracts from B-13 cells transfected with the indicated construct. Each lane contains 10 μg protein/lane (Left Panel). Right Panel, B-13 cells transfected as indicated with pcDNA empty vector or pcDNA-LPLA2 as outlined in the Methods section and after 24 h the cells were treated with indicated doses of tamoxifen and additionally incubated with LipidTOX (red). After a further 24 h, the cells were fixed and stained with DAPI (blue) and phospholipidosis quantified and normalised to DAPI fluorescence as outlined in the Methods section, *significantly different phospholipidosis (two tailed) from cells transfected with pcDNA-empty using the Student’s *T* test (p < 0.05). C, B-13 cells were transfected with indicated concentrations of pcDNA-LPLA2 and RL-TK to a final concentration of 2 μg per well as outlined in the Methods section and after 24 h, the cells were treated with 6 μM tamoxifen. After a further 24 h, phospholipidosis and renilla activities were determined (separate samples). Significantly different *phospholipidosis or $renilla activity (two tailed) versus cell transfected in the absence of LPLA2 or RL-TK respectively using ANOVA (p < 0.05) followed by Bonferonni post hoc test. (For interpretation of the references to colour in this figure legend, the reader is referred to the web version of this article.)

Fig. 4A confirms that B-13 and B-13/H cells express Lpla2 transcripts similarly to liver and many other tissues and therefore – assuming translation to protein – the cells are unlikely to be sensitized to phospholipidosis due to the absence of functional Lpla2. Fig. 4B demonstrates that ectopic expression of human LPLA2 with phospholipidosis-inducing concentrations of tamoxifen in B-13 cells resulted in a statistically significant reduction of phospholipidosis, supporting a role for a block of in LPLA2 interaction with lysosomal membranes in phospholipidosis. This effect was LPLA2-dependent at a fixed concentration of tamoxifen, such that transfecting increasing amounts of LPLA2-encoding plasmid into B-13 cells resulted in a dose-dependent reduction in phopsholipidosis (Fig. 4C).

### 3.5 Methapyrilene induces phospholipidosis in B-13 and B-13/H cells

**Fig. 5:**
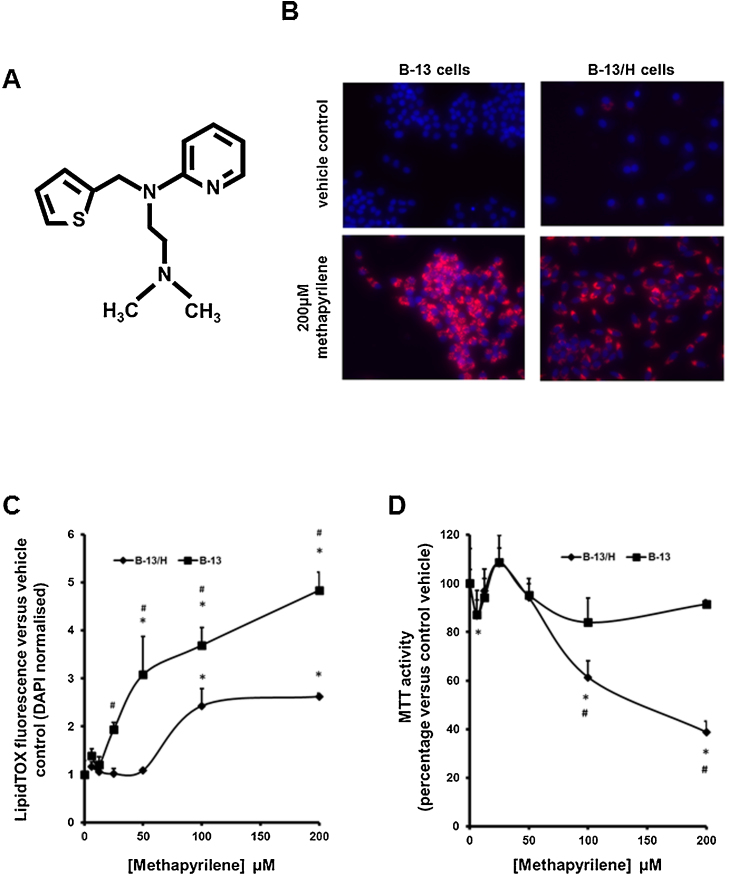

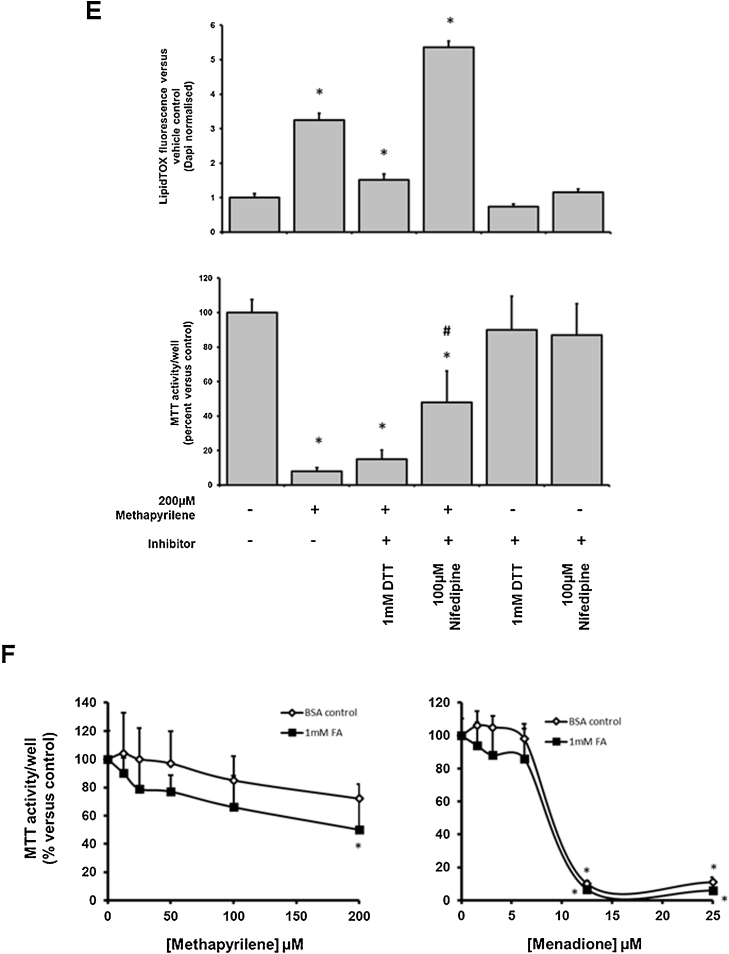
Methapyrilene exposure causes phospholipidosis in B-13 and B-13/H cells. A, structure of methapyrilene. B, B-13 or B-13/H cells were treated once for 2 days with medium supplemented with methapyrilene. After 24 h, cells were additionally incubated with LipidTOX (red) and at 48 h cells were fixed and stained with DAPI (blue) prior to fluorescence imaging as outlined in Methods section. C, B-13 or B-13/H cells were treated once for 2 days with medium supplemented with the indicated concentration of drugs. After 24 h, cells were additionally incubated with LipidTOX (red) and at 48 h cells were fixed and stained with DAPI (blue) and phospholipidosis quantified and normalised to DAPI fluorescence as outlined in Methods section. Data are the mean and standard deviation of 3 separate determinations from the same experiment, typical of 3 separate experiments, significantly different phospholipidosis (two tailed) from *control treated cells using ANOVA (p < 0.05) followed by Bonferonni post hoc test or #versus B-13 cells treated with the same concentration of methapyrilene using the Student’s *T* test (two tailed) (p < 0.05). D, B-13 and B-13/H cells treated as in C. After 46 h, cells were incubated with MTT for 2 h and reduction determined as outlined in Methods section prior to protein determination. Data are the mean and standard deviation MTT activity normalised to cell protein of 3 separate determinations from the same experiment, typical of 2 separate experiments, significantly different MTT activity (two tailed) from *control treated cells using ANOVA (p < 0.05) followed by Bonferonni post hoc test or #versus B-13 cells treated with the same concentration of methapyrilene using the Student’s *T* test (two tailed) (p < 0.05). E, Upper panel – B-13/H cells were pre-incubated with indicated treatment for 4 h and then re-treated with where indicated, 200 μM methapyrilene, for 24 h. The cells were then incubated with LipidTOX (red) and at 48 h, cells were fixed and stained with DAPI (blue) and phospholipidosis quantified and normalised to DAPI fluorescence as outlined in Methods section. Lower panel – B-13/H cells were pre-incubated as above and at 46 h, cells were incubated with MTT for the final 2 h and reduction determined as outlined in the methods section. Data are the mean and standard deviation MTT activity normalised to cell protein of 3 separate determinations from the same experiment, typical of 2 separate experiments. Significantly different (two tailed) phosholipidosis versus *control treated or #methapyrilene only treated cells using ANOVA (p < 0.05) followed by Bonferonni post hoc test. F, B-13/H cells were pre-treated with BSA or 1 mM fatty acids for 24 h to induce steatosis, washed and then incubated methapyrilene or menadione for 24 h in normal medium. Cells were incubated with MTT for 2 h and reduction determined as outlined in the Methods section. Data are the mean and standard deviation MTT activity normalised to cell protein of 5 separate determination from the same experiment, typical of 3 separate experiments. *Significantly different (two tailed) MTT activity versus control treated cells using ANOVA (p < 0.05) followed by Bonferonni post hoc test. (For interpretation of the references to colour in this figure legend, the reader is referred to the web version of this article.)

Methapyrilene is a H1 histamine antagonist that was withdrawn from the market because it was shown to be a non-genotoxic rat liver carcinogen (Habs et al., 1986). Methapyrilene is also toxic to rat hepatocytes in vitro (Ratra et al., 1998a) and induces periportal liver injury when orally administered to rats (Ratra et al., 1998b). Methapyrilene has a cationic amphiphilic structure (Fig. 5A) but has not, to our knowledge, previously been investigated for its ability to induce phospholipidosis.

Fig. 5B and C demonstrate that methapyrilene exposure resulted in phospholipidosis in both B-13 and B-13/H cells in dose-dependent manners, most potently in B-13 cells. As previously reported (Probert et al., 2014), methapyrilene was only toxic to B-13/H cells and not to B-13 cells and similar results were observed in these studies (Fig. 5D). Previous work has also demonstrated that methapyrilene hepatoxicity is inhibited by sulfhydryl reducing agents and the Ca^2+^ channel blocker nifedipine (Ratra et al., 1998a, Probert et al., 2014). Fig. 5E demonstrates that methapyrilene-induced phospholipidosis in B-13/H cells was increased when cells were co-incubated with nifedipine but decreased when co-incubated with dithiothreitol (DTT). However, when phospholipidosis was reduced through DTT – or increased through nifedipine additions – methapyrilene toxicity was unaffected or significantly decreased respectively (Fig. 5, lower panel). In contrast, no significant modulating effects were observed when steatotic B-13/H cells were treated with methapyrilene (Fig. 5F left panel) and also with menadione, a toxin that does not cause lipid dysregulation (Fig. 5F, right panel).

Since B-13 cells develop significant phospholipidosis after methapyrilene exposure but do not undergo cell death and B-13/H cell death is inhibited by nifedipine but phospholipidosis is increased, these data suggest that phospholipidosis is not a critical event in methapyrilene-induced hepatotoxicity. Interestingly, addition of DTT to B-13/H cells treated with amiodarone or tamoxifen potently blocked the induction of phospholipidosis (Supp Fig. S4), suggesting that an oxidative redox status and/or protein thiol oxidation is necessary for phospholipid accumulation. Addition of DTT to cultures after induction of phospholipidosis did not affect the fluorescence readout, confirming that the effect of DTT was cell-dependent and not associated with a direct chemical interaction with the detecting fluorophore (e.g. through fluorescence quenching), data not shown.

Fig. S4 DTT treatment inhibits cationic amphiphilic drug-induced phospholiposis in B-13/H cells. B-13/H cells were pre-incubated with 2 mM DTT for 4 h followed by treatment with the indicated phospholipidosis-inducing drugs. At 24 h, cells were additionally incubated with LipidTOX (red) and at 48 h cells were fixed and stained with DAPI (blue) and phospholipidosis quantified and normalised to DAPI fluorescence as outlined in Methods section. *Significantly different (two tailed) phospholipidosis versus cells treated in the absence of DTT using the Student’s *T* test (p < 0.05).

## 4 Discussion

The B-13 cell line is a unique tool for generating hepatocyte-like B-13/H cells using a simple protocol and in a cost-effective manner. Expression of many drug metabolising enzymes such as cytochrome P450 s and conjugating enzymes at levels similar to those present in intact rat liver remains a prominent feature and has recently been exploited in toxicity and genotoxicity studies (Marek et al., 2003, Wallace et al., 2010c, Probert et al., 2015). Many of the drug metabolising enzymes are functional under the simple (e.g. plastic substrata, 2D format, no requirement for recombinant factors) culture methodology employed in the current study though undoubtedly increased functionality and utility in toxicological studies is likely through modification of the medium components (e.g. addition of haem precursors to bolster functional cytochrome P450 levels (Fairhall et al., 2013a)). However, little attention has been paid to these aspects to date.

This paper demonstrates for the first time that B-13 cells are homozygous wild type for the PNPLA3 gene, for which a polymorphism for susceptibility to NAFLD in man has been identified (Liu et al., 2014). Nonetheless, B-13/H cells will accumulate intracellular triglycerides in the form of lipid droplets (steatosis) in response to exposure to high levels of medium fatty acids (bound to albumin to reduce their toxicity) or when exposed to the LXR activator T0901317. Storage of neutral lipids has been shown to be directly proportional to the abundance of ADFP, a protein residing at the surface of lipid droplets and indicated to be involved in the metabolism of stored lipids (Chang et al., 2006). Since GW3965 induces ADFP in human but not in rat hepatocytes, this may explain why GW3965 activated the LXR in B-13/H cells, but did not markedly promote steatosis. Interestingly, the B-13 progenitor cell does not become markedly steatotic in response to fatty acid exposure. The processes of fatty acid uptake by cells remain to be fully defined. Despite the presence in some cells of protein transporters, diffusion remains one route for fatty acids to enter cells. The lack of steatosis in B-13 cells in response to either fatty acids or T0901317 may be associated with a lack of CD36 and LXR expression respectively. That B-13/H cells respond to these challenges by becoming steatotic however, serves to underpin their functionality as hepatocyte-like, since this is a response observed in hepatocytes in vivo to these challenges. In addition, we report for the first time that the both B-13 and B-13/H cells accumulate phospholipids (phospholipidosis) when exposed to hydrophobic amphipathic drugs and that the mechanism at least in part, is associated with an inhibition and/or degradation of LPLA2.

In all cases, the dysregulated accumulation of lipids (steatosis or phospholipidosis) show time and dose-response effects.

Given the regulatory requirements for drug and chemical safety testing (e.g. the Registration, Evaluation, Authorisation & restriction of Chemicals/REACH), in vitro platform technologies may be the only practical way to estimate potential adverse effects in man. A major hurdle to overcome in exploiting these technologies will be to progress from confirming that a technology correctly predicts known adverse effects, to accepting that a technology will predict adverse effects of a chemical in vivo. In this respect, methapyrilene was examined for its ability to induce phospholipidosis. Methapyrilene was selected because the liver is a target organ for toxicity to this drug, its chemical structure includes moieties that are associated with phospholipidosis induction and because, to our knowledge, this effect had not been previously examined. We demonstrate that methapyrilene is a potent inducer of phospholipidosis but that this response likely plays a minimal role in necrosis since induction is greater in B-13 cells, which were insensitive to methapyrilene toxicity. Furthermore, compounds which blocked methapyriline toxicity in B-13/H cells also increased the accumulation of phospholipids. Given the simplicity of generation, this paper demonstrates that B-13/H cells are an effective model cell system in which to study hepatic lipid dysregulation in response to elevated fatty acids, LXR activators and chemicals capable of inducing phospholipidosis.

## Conflict of interest

None.

## Funding

A.C.L is supported by an ITTP studentship award from the Medical Research Council. This work was also supported in part by grants from the European Foundation for Alcohol Research (grant no. EA1402); Alternatives Research and Development Foundation (http://www.ardf-online.org/) and by European Commission FP7 program grant‘D-Liver’ (EC Contract No. 287596; http://www.D-LIVER.eu/).
