# Supplementary figures and images for: B-13 progenitor-derived hepatocytes (B-13/H cells) model lipid dysregulation in response to drugs and chemicals
Source: Toxicology. 2017 Jul 1;386:120–32. doi: 10.1016/j.tox.2017.05.014 (PMC5553091; doi:10.1016/j.tox.2017.05.014)

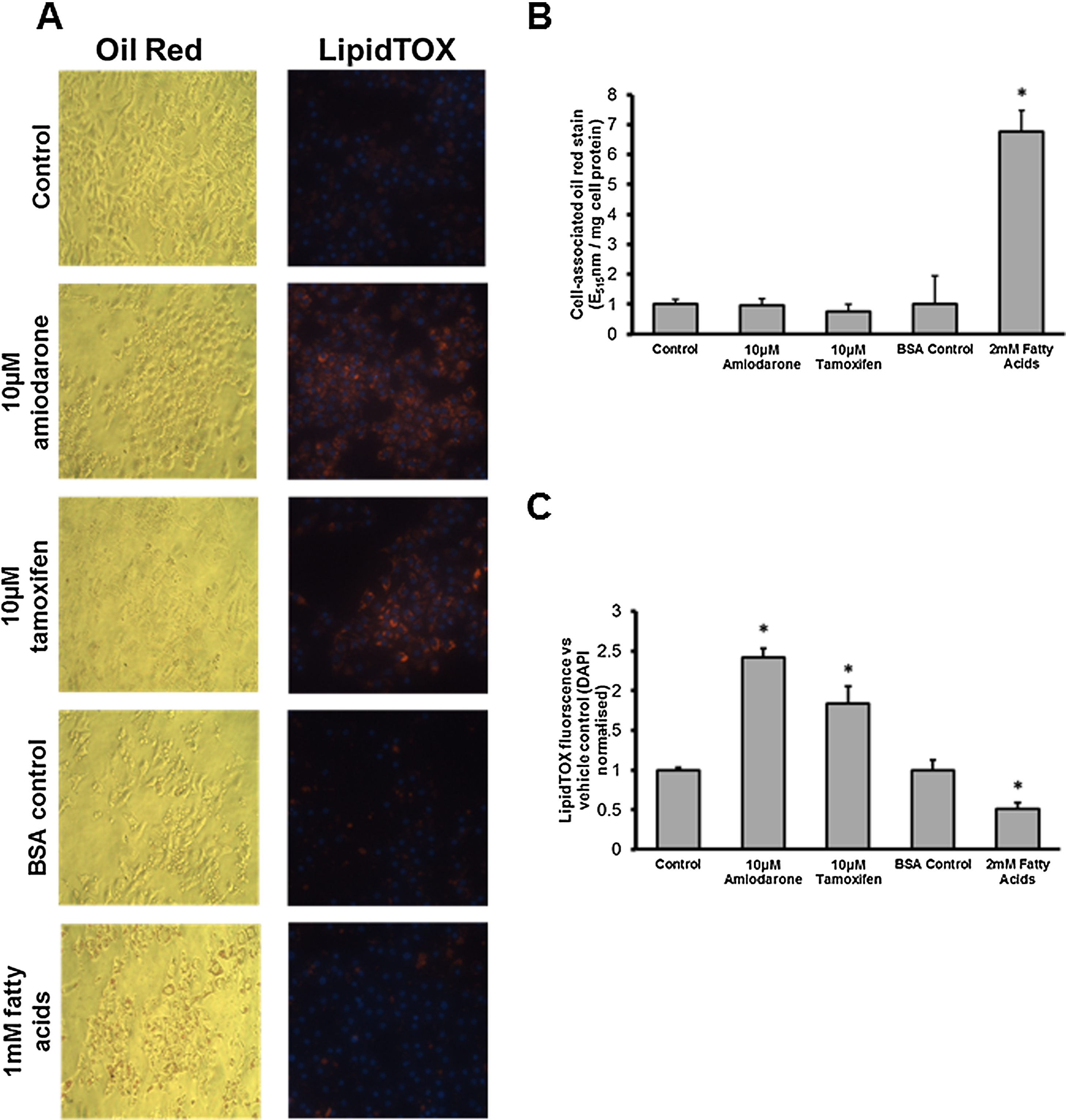

Supplement: Fig. S1 — Specificity of oil red and LipidTOX stains for steatosis and phospholipidosis respectively. B-13/H cells were untreated (control) or treated daily for 2 days with medium supplemented where indicated with 2 mM fatty acids, its vehicle control (BSA control) or at the indicated concentration of selected amphiphilic drugs and incubated for the detection of steatosis or phospholipidosis as outlined in the Methods section. Twenty four hours prior to fixation and analysis, cells for phospholipidosis detection were additionally incubated with LipidTOX. Cells were fixed and either stained with oil red or stained with DAPI and imaged (A) and then steatosis lipid accumulation quantified by extraction of oil red from stained cells and determination of absorbance at 515 nm (B) or LipidTOX and DAPI fluorescence determined (C). Data are the mean and standard deviation of 3 separate determinations from the same experiment, typical of 3 separate experiments, *significantly different (two tailed) from control treated cells using ANOVA (p < 0.05) followed by Bonferonni post hoc test. [file mmc2.jpg]

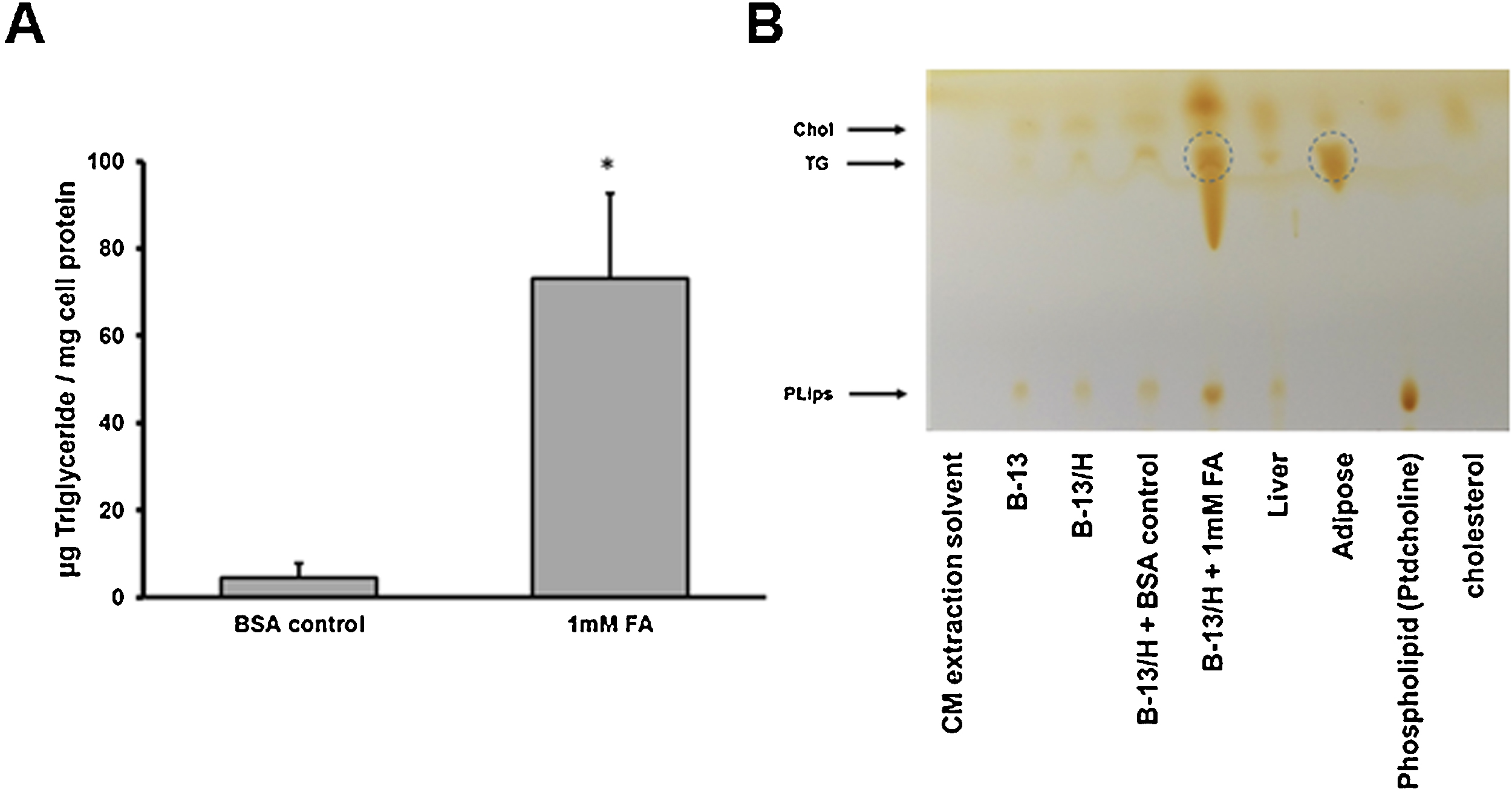

Supplement: Fig. S2 — Triglyceride accumulation in B-13/H cells. A, triglyceride content in B-13/H cells after incubation with 1mM fatty acids for 3 days. B, TLC analysis of lipids extracted from the indicated cells/tissue (triglycerides indicated by dotted circles; Ptdcholine = phosphatidylcholine). [file mmc3.jpg]

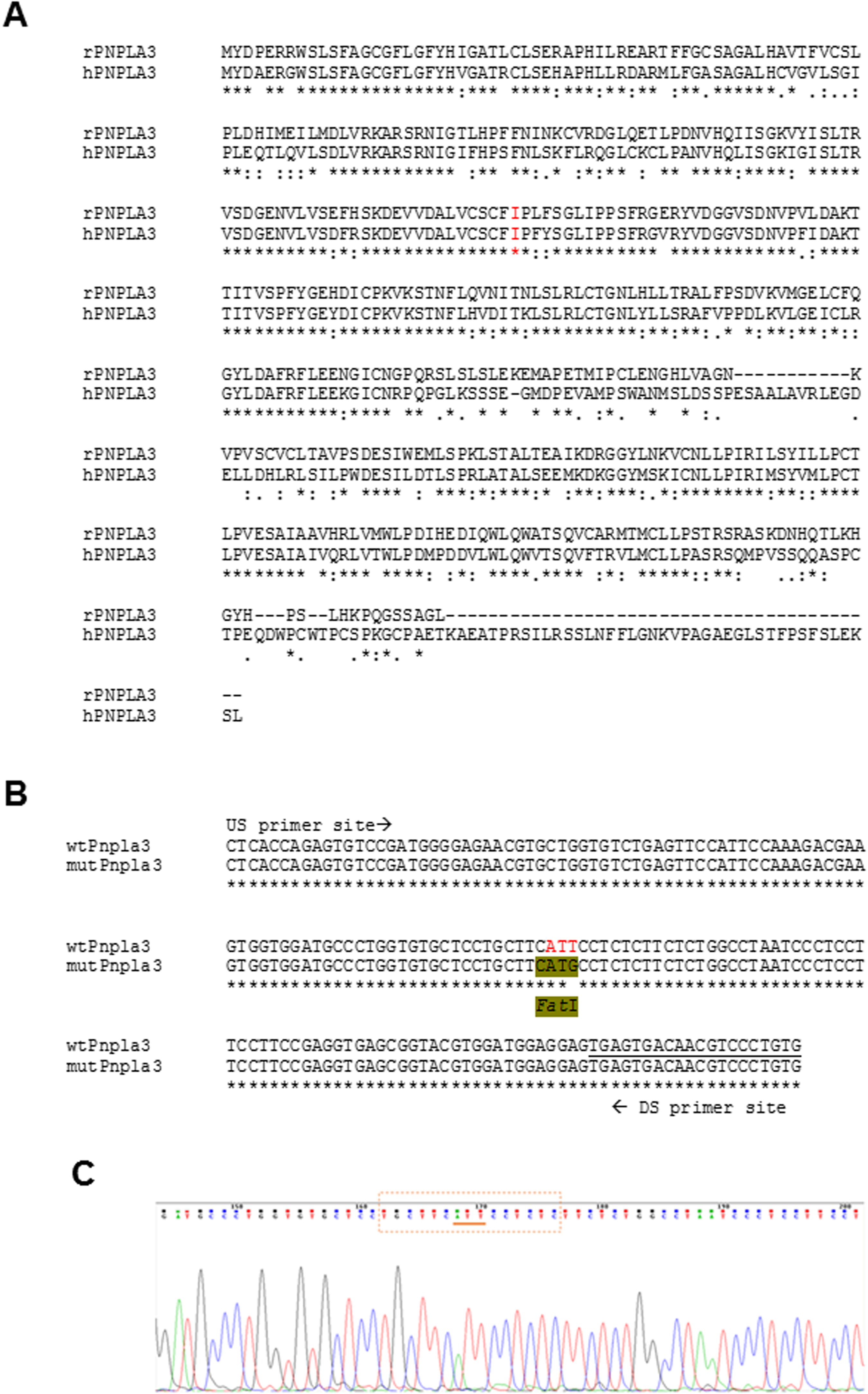

Supplement: Fig. S3 — The B-13 cells is homozygous wild type for the patatin-like phospholipase domain-containing protein 3 (Pnpla3) gene. A, CLUSTAL O (1.2.1) multiple sequence alignment (http://www.ebi.ac.uk/Tools/msa/clustalo/) of rat (rPNPLA3) and human (hPNPLA3) amino acid sequences, with the wild type isoleucine residue at position 148 indicated in red. “*”, a single, fully conserved residue; “:”, conservation between groups of strongly similar properties; “.”, conservation between groups of weakly similar properties; – no residue alignment. B, Alignment of CLUSTAL O (1.2.1) multiple sequence alignment of the wild type rat Pnpla3 cDNA sequence (wtPnpla3) and the predicted mutant Pnpla3 cDNA sequence (mutPnpla3) amplified by RT-PCR using the upstream (US) and downstream (DS) primers as indicated. Note, ATT codes for isoleucine (I) whereas ATG codes for methionine (M). Note, both ATC and ATA codons code for I and therefore the wild type protein. Therefore, restriction of PCR products with the endonuclease FatI at this site indicates the existence of a mutant transcript, which in man, is strongly associated with NAFLD incidence (Anstee QM, Seth D, Day CP. Genetic Factors That Affect Risk of Alcoholic and Nonalcoholic Fatty Liver Disease. Gastroenterology. 2016 Jun;150:1728–1744). C, Typical sequencing data from RT-PCR product amplifying the region of the B-13 patatin-like phospholipase domain-containing protein 3 (PNPLA3) cDNA sequence. [file mmc4.jpg]

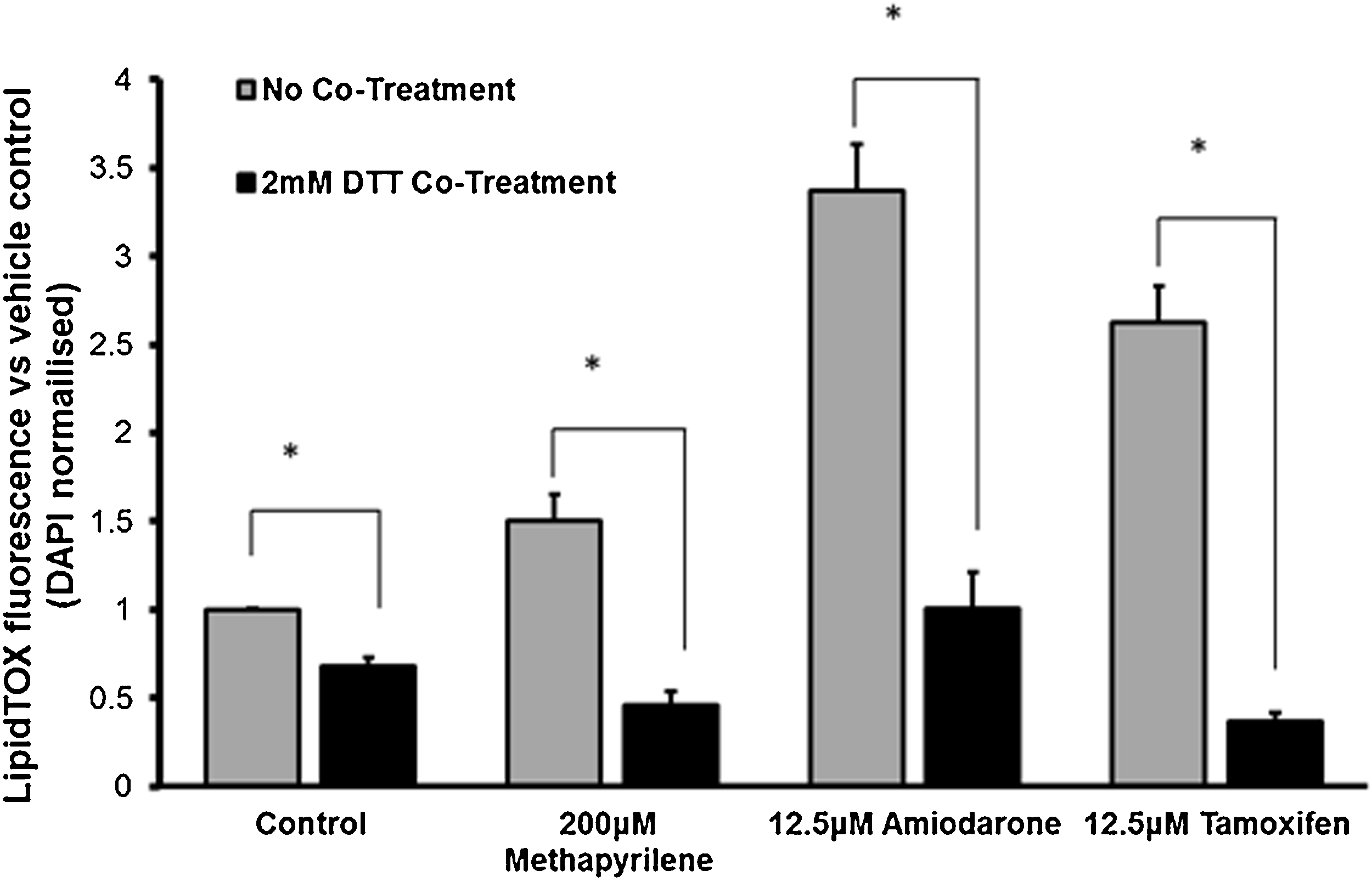

Supplement: Fig. S4 — DTT treatment inhibits cationic amphiphilic drug-induced phospholiposis in B-13/H cells. B-13/H cells were pre-incubated with 2 mM DTT for 4 h followed by treatment with the indicated phospholipidosis-inducing drugs. At 24 h, cells were additionally incubated with LipidTOX (red) and at 48 h cells were fixed and stained with DAPI (blue) and phospholipidosis quantified and normalised to DAPI fluorescence as outlined in Methods section. *Significantly different (two tailed) phospholipidosis versus cells treated in the absence of DTT using the Student’s T test (p < 0.05). [file mmc5.jpg]
