# Supplementary material for: B-13 progenitor-derived hepatocytes (B-13/H cells) model lipid dysregulation in response to drugs and chemicals
Source: Toxicology. 2017 Jul 1;386:120–32. doi: 10.1016/j.tox.2017.05.014 (PMC5553091; doi:10.1016/j.tox.2017.05.014)
Supplement: Table S1 [file mmc1.doc]

**Supplementary Table 1: Primers used for RT-PCR**

| **Oligo ID** | **Primer sequence (5'-3')** | | **Comments** |
| --- | --- | --- | --- |
| **Fatty acid uptake** | | | |
| **rCD36** | **US** | TCCTCGGATGGCTAGCTGATT | Will amplify 187bp fragment from rat CD36 molecule / thrombospondin receptor (NM_031561.2) cDNA. |
|  | **DS** | GCACTTGCTTCTTGCCAACT |
| **rSlc27a1** | **US** | CTGCGAGAACCCGTGAGGAA | Will amplify 166bp fragment from rat solute carrier family 27 (fatty acid transporter) Slc27a1 (NM_053580.2) cDNA. |
|  | **DS** | ACCCACGTACACACCGAAC |
| **rSlc27a2** | **US** | GAATGTTTACGGTGTGCCCG | Will amplify 123bp fragment from rat solute carrier family 27 (fatty acid transporter) Slc27a2 (NM_031736.1) cDNA. |
|  | **DS** | GGCAGGTACTCCGAGATGTG |
| **rSlc27a4** | **US** | GCAACTGTAGCTTGGGCAAC | Will amplify 87bp fragment from rat solute carrier family 27 (fatty acid transporter) Slc27a4 (NM_001100706.1) cDNA. |
|  | **DS** | AGCGGATGGGGTACACAAAG |
| **rSlc27a5** | **US** | CATTCGATGGGGCTTGTCCT | Will amplify 122bp fragment from rat solute carrier family 27 (fatty acid transporter) Slc27a5 (NM_024143.2) cDNA. |
|  | **DS** | ACTGTCACACTGTACTGCCG |
| **Acetyl-CoA synthetases** | | | |
| rAcsl | US | GCAGGGGTGCTTCACTTACT | Will amplify 249bp fragment from predicted rat ) acyl-CoA synthetase long-chain family member 1 transcript variant X6 Acs1 (XM_006253125.2) cDNA and 7 other transcript variants. |
|  | DS | CCGATGATTTCCACCCCACA |
| rAcs4 | US | CTACTGGAAGAGTTGGCGCT | Will amplify 301bp fragment from predicted rat ) acyl-CoA synthetase long-chain family member 4 Acs4 transcript variant X4 (XM_006257316.2) cDNA and 4 other transcript variants. |
|  | DS | GACACGTACTCTCCGGCTTG |
| rAcs5 | US | TGACCCCAAAGGAGCTATGC | Will amplify 134bp fragment from rat Acs5 (NM_053607.1) cDNA. |
|  | DS | ACATATGGGCCAAGGGAAGG |
| **Acetyl-CoA carboxylases** | | | |
| rAcaca | US | GGGAACATCCCCACGCTAAA | Will amplify 126bp fragment from rat acetyl-CoA carboxylase alpha Acaca (NM_022193.1) cDNA. |
|  | DS | CATGCGTTGACAAGGTGGTG |
| rAcacb | US | GGTCCTGATTGCCTCTCACC | Will amplify 197bp fragment from rat acetyl-CoA carboxylase beta Acacb (NM_053922.1) cDNA and 6 other transcript variants. |
|  | DS | ACGCCATACAGACGACCTTG |
| **ATP citrate lyase** | | | |
| rAcly | US | ATCCGGGGAGTTGGGGTAAG | Will amplify 125bp fragment from rat ATP citrate lyase Acly transcript variant 2 (NM_001111095.1) cDNA and also transcript variant 1. |
|  | DS | CTTTGCCGGTCTGCTCTGAA |
| **Mitochondrial transport** | | | |
| rMpc1 | US | ATGAGTACGCACTTCTGGGG | Will amplify 213bp fragment from rat mitochondrial pyruvate carrier Mpc1 (NM_133561.1) cDNA. |
|  | DS | AATGAGCTGAGCGACTTCGT |
| rMpc2 | US | GTGCTGATGGCTACAGGGTT | Will amplify 163bp fragment from rat mitochondrial pyruvate carrier Mpc2 (NM_001077643.1) cDNA |
|  | DS | ACTGGATTCCTTTAGATTTGAGTTC |
| rSlc25a1 | US | CCAAGGAGACAACCCCAACA | Will amplify 255bp fragment from rat mitochondrial citrate carrier Slc25a1 (NM_017307.3) cDNA |
|  | DS | AATACGATGGCCACGTCCAG |
| **Fatty acid synthase** | | | |
| rFasn | US | CCCTCACATCAAGTGGGACC | Will amplify 387bp fragment from rat fatty acid synthase (NM_017332.1) cDNA. |
|  | DS | TGGTACACTTTCCCGCTCAC |
| **Lipid storage / metabolism** | | | |
| rPlin1 | US | GTGGCTCTCAGCTGCATGT | Will amplify 87bp fragment from rat perilipin 1 Plin1 (NM_001308145.1) cDNA. May also amplify variant X1 (XM_008759499.1) yielding a 143bp fragment although DS primer has 3 MM. |
|  | DS | ATTCTCCTGTTCAGGGAGGTCT |
| rPlin2 | US | CAGTACTTGCCGCTCACTCA | Will amplify 240bp fragment from rat perilipin 2 (Adrp) Plin2 (NM_001007144.1) cDNA, intrinsic lipid storage droplet protein. Also amplifies X1 and X3 transcript variant cDNAs. |
|  | DS | GTGCACATTCTTCCTGGCGAA |
| **rPlin3** | **US** | AGCAGTGGATGTGACCTGTG | Will amplify 202bp fragment from predicted rat Plin3 (XM_001061015.2) cDNA. |
|  | **DS** | TGTGGCAATCAGGGCTAGTT |
| **rPlin4** | **US** | ATGGGACTAGAGGTTCCCCC | Will amplify 166bp fragment from 47 predicted rat Plin4 transcript variants (e.g. XM_006244382.2) cDNA. |
|  | **DS** | GGAGATGGAGGGACAGGAGT |
| **rPlin5** | **US** | GCTCTACACAGCAGGATGTCCG | Will amplify 534bp fragment from rat Plin5(NM_001134637.1) cDNA. Will also amplify transcript variant X1 (XM_008766770.1) yielding a 498bp fragment. |
|  | **DS** | CAGCTCCTCTGATTTGCCCAG |
|  |  |  |  |
